# Hock lesions in dairy cows in freestall herds: a cross-sectional study of prevalence and risk factors

**DOI:** 10.1186/s13028-018-0401-9

**Published:** 2018-08-13

**Authors:** Lisa Ekman, Ann-Kristin Nyman, Håkan Landin, Karin Persson Waller

**Affiliations:** 1 Department of Animal Health and Antimicrobial Strategies, National Veterinary Institute, 75189 Uppsala, Sweden; 2 Department of Clinical Sciences, Swedish University of Agricultural Sciences, 75007 Uppsala, Sweden; 3 Växa Sverige, 10425 Stockholm, Sweden

**Keywords:** Dairy cow, Epidemiology, Hock damage, Injury, Pressure ulcer, Skin lesion, Sweden

## Abstract

**Background:**

Hock lesions (HL) in dairy cows are a common animal welfare problem in modern dairy production with freestall housing systems, but there are no large-scale studies addressing its epidemiology in Sweden. The aims of this cross-sectional study were to investigate the prevalence of HL of different severity in 100 Swedish freestall dairy herds, and to identify cow- and herd-related risk factors. Associations between HL and mastitis as well as culling were also investigated.

**Results:**

In total, 3217 cows from 99 herds were included in the statistical analyses. The overall cow prevalence of hair loss on the hock (mild HL) was 68% and the prevalence of ulceration or evident swelling of the hock, with or without hair loss, (severe HL) was 6%. The within-herd prevalence varied among herds, between 23 and 100% for mild HL, and between 0 and 32% for severe ones. Breed (higher risk for Swedish Holstein than for Swedish Red) and days in milk (higher risk at 181–305 days than at 0–90 days) were cow-related risk factors associated with both types of lesions, whereas higher parity and cleaner cows were associated only with increased risk of severe HL. A reduced risk for mild HL was seen in cows housed on mattresses compared to rubber mats, and in cows housed on peat compared to other bedding materials. Also, cows in herds with a high proportion of not yet inseminated heifers older than 17 months had a lower risk of mild HL than cows in herds with a low proportion. Risk for severe HL was lower when cubicles were of recommended width compared to under recommendation, for organic production compared to conventional, and when teat dip or no treatment after milking was used, compared to teat spray. For both mild and severe HL, herringbone milking parlors were associated with higher risk than tandem parlors. We found no significant associations between HL and mastitis or culling.

**Conclusions:**

The prevalence of HL is high in Swedish dairy herds, although most lesions are mild. Several cow- and herd-related risk factors were identified and the results can be used to improve recommendations for the prevention of HL in Swedish freestall dairy herds.

**Electronic supplementary material:**

The online version of this article (10.1186/s13028-018-0401-9) contains supplementary material, which is available to authorized users.

## Background

During recent decades, the dairy industry in the Scandinavian countries has undergone marked structural rationalization resulting in increased milk production, larger herds and an increasing proportion of freestall housing systems [1, 2]. Freestalls facilitate natural behavior and several Nordic countries legislate such housing systems to improve animal welfare [3, 4]. However, the introduction of new housing systems is a challenge for dairy producers, as inadequate management or freestall design may lead to welfare problems such as lameness and skin lesions [5–7]. Hock lesions (HL) are a common problem in modern dairy production, and several studies have investigated the epidemiology of HL in freestall dairy herds [3, 8–14]. Similar to pressure ulcers in bedridden human patients, these lesions may develop due to prolonged pressure on soft tissues between bony prominences and the underlying surface, causing reduced blood flow, lack of oxygen, and tissue necrosis in the affected area [15]. Hock lesions may also result from direct trauma to the hock, caused by abrasive lying surfaces or other physical conflicts between the cow and her surroundings [3, 9]. Presence of HL can be painful for affected animals and is also associated with lameness [9, 16] and high culling rates [6, 17]. Moreover, HL might have a negative impact on udder health [6, 18].

As there is no widely adapted definition of HL the scoring of lesions differs between studies, which makes comparisons of results difficult [11]. The most common, and mildest, form of HL is localized hair loss on the lateral aspect of the hock [8, 19] while more severe lesions with swelling of the hock region or skin ulceration are seen less often. Different symptoms of HL (e.g. hair loss, swelling, and ulceration) may occur separately or in combination with each other, and the underlying cause may differ depending on the type of lesion [8, 16]. Despite the variation in scoring systems, the prevalence of HL in freestalls is high in several studies, ranging from 50 to 87% [3, 8–10]. There is a lack of large-scale Swedish studies on HL epidemiology, especially in freestalls, although one study investigated HL prevalence and risk factors in 55 Swedish dairy herds (that included 18 freestall herds) [20]. The observed prevalence was 30%, with a higher risk for cows housed in freestalls than tie-stalls [21].

The complex relationships between individual cow factors, housing- and management factors, and HL are not fully understood. Moreover, risk factors may differ depending on the type of HL [16]. This indicates that mild and severe HL should be modelled separately.

Previous studies from other countries have found risk factors for HL, including higher parity [3, 17], increased milk yield [16], and Holstein breed [16]. Other factors that affect the occurrence of HL include housing- and management-related factors such as the production system (organic or conventional) [3, 17]; bedding material [17, 22]; and cubicle size [3, 16, 23]. However, results from previous studies are not uniform and even contradictory for some risk factors (e.g., herd size [16, 17], and cow hygiene [16, 22]).

As dairy production conditions differ between regions and countries, e.g. depending on legislation, traditions and climate, risk factors for HL might differ even though similar production systems are used. This warrants investigating risk factors for different types of HL in Swedish dairy production conditions, as there are very few such studies performed. In addition, HL is important to study because it has been associated with high bulk-tank somatic cell count (SCC) [6] and clinical mastitis [18], which might lead to impaired animal welfare as well as production losses for the farmer.

Our aims were to investigate the prevalence of HL of different severity in Swedish freestall dairy herds, and to identify cow- and herd-related risk factors. Identification of HL risk factors is necessary to improve recommendations on how to prevent such lesions. The associations between HL and mastitis and culling were also investigated.

## Methods

### Herd selection and data collection

Herd selection and data collection were performed as described in Ekman et al. [24]. Briefly, 100 randomly selected Swedish dairy herds with freestall housing, herd size of 50–210 cows, and affiliation with the Swedish Official Milk Recording Scheme (SOMRS) were enrolled in a cross-sectional study. Each herd was visited during one milking in the winter housing season, i.e., February–April 2014 (39 herds) or December–March 2014–2015 (61 herds). All visits and registrations were performed by the first author. Additional cow and herd data were obtained from the SOMRS.

### Cow data

A detailed list of cow-related variables (n = 16) and how they were obtained is given in Additional file 1. At the herd visit, a random number of cows (every second to third cow to enter the milking parlor depending on the speed of the milking process) was examined for HL and assigned a hygiene score. For practical reasons, the presence of HL was assessed on the lateral side of the hock visible from the operating area of the milking parlor, thus, one hock per cow was examined. A HL was registered as mild when there was loss of hair (regardless of the size of the hairless area) and as severe when there was evident swelling and/or ulceration in the hock area, with or without hair loss, according to the Hock Assessment Chart for Cattle [25]. Cow hygiene scores were based on the cleanliness of the udder and the hind limb above the hock visible from the operating area. Scores ranged from 1 to 4, where 1 was completely clean; 2 indicated manure stains on hind limb and/or udder; 3 was one to two areas of manure patches of at least 10 cm in diameter; and 4 was more than two areas of manure patches as described for score 3 (modified from Cook [26]).

Information on breed, parity and days in milk (DIM) for individual cows at the time of the visit was obtained from the SOMRS, as was information on individual milk yield [kg energy corrected milk (ECM)/day], cow composite milk somatic cell count (SCC), and milk urea levels from test milkings within 34 days before or after the visit. Registrations of veterinary-treated diseases and results from hoof trimmings (when available within 90 days before or after the visit) were collected from the SOMRS, as well as registrations on culling within 90 days post-visit. The 90 day limit was set to avoid investigating events too distant in time from the registered HL.

### Herd data

The list of collected herd-related variables and how they were obtained is given in Additional file 2. Table 1 presents an overview of these herd-related variables, grouped into general herd factors, housing-related factors, management-related factors, and herd health-related factors. Milking routines, housing-related factors, and other management-related factors were registered while at the farms, through observations or interviews with the farm owner or staff. Additional herd data were obtained from the SOMRS, including herd performance indicators, for the 12 months preceding the herd visit. These herd performance indicators, designed by Växa Sverige (a Swedish organization for dairy farmers, Stockholm, Sweden), include calf, young stock and cow mortality; incidence of culling and veterinary-treated diseases; and percentage of cows with abnormal milk urea levels and fertility traits.

**Table 1:** Herd-related variables analyzed for associations with hock lesions (HL) in 99 Swedish dairy herds

Group of variables	Number of variables	Examples of variables
General herd factors	4	Herd size (cows/year), production system, milk production, slaughter weights
Housing-related factors	17	Cubicle dimensions, year of cubicle installation, cubicle base, bedding material, stocking ratio (cows/cubicle)
Management -related factors	18	Pasture period, cubicle and alley cleaning, hoof trimming, milking, and feeding routines
Herd health-related factors	27	Presence of ectoparasites and digital dermatitis, calf and cow mortality^a^, incidence rate of cullings and veterinary-treated diseases^a^, fertility traits^a^

^a^Based on “Welfare signals”—herd performance indicators from the Swedish Official Milk Recording Scheme (Växa Sverige, Stockholm) based on the 12 months preceding the herd visit

### Data editing and statistical analyses

Cow and herd data registered in handwritten protocols were transferred to Excel sheets. The data were imported and analyzed with Stata (release 13.1; StataCorp LP, College Station, TX, USA) after verification by an additional person that the transfer of data from the protocols was correct. The prevalence of mild, severe, and all HL was calculated for all examined cows, and within each herd.

### Risk factors for mild and severe HL

Associations between the presence of mild or severe HL and the explanatory variables were analysed with univariable mixed-effect logistic regression or mixed-effect linear regression models. The analyses were done at cow-level (both for cow and herd related risk factors), with herd as a random factor using an identity covariance structure (equal variances for random effects; all covariances are zero). Continuous variables were assessed if they were linearly related to the outcome. Where they were not, they were categorized using percentiles as cut-offs, or transformed using the natural logarithm. Cows with severe HL were excluded from the analyses with mild HL as dependent variable and vice versa. In total, 15 cow-related factors and 66 herd-related ones were analyzed for their association with mild and severe HL. All variables with a *P*-value of 0.20 or less were kept for further analyses in multivariable mixed-effect logistic regression models with herd as random factor.

Due to the large number of independent variables, four separate multivariable mixed-effect logistic regression sub-models were constructed for each of the outcomes (mild or severe HL). Sub-model 1 included cow-related factors (Additional file 1) and sub-model 2–4 included herd-related factors divided into three categories: general herd and housing, management, and herd health (Additional file 2). Spearman rank correlations were used to test collinearity between variables in all multivariable models. If two variables showed collinearity (r ≥ 0.7), the one with the lowest *P*-value was kept in the model. Models were then built using a manual, stepwise backward variable-selection procedure where the initial model included all independent variables as main effects. Variables with a significant association (*P* ≤ 0.05) with the dependent variable were kept in the models. Visit period (February–April 2014 or December–March 2014–2015) was included in all multivariable models as a potential confounder. Cows with missing data for a specific independent variable were omitted when that variable was in the model. Two herds left the SOMRS shortly before or after the visit and were therefore excluded in the multivariable analyses due to missing data. If less than 5% of cows belonged to a specific category within a variable, that category was removed from the multivariable analyses. In addition, variables with many missing values (> 30% missing data) were not used in the multivariable analyses.

A final, multivariable mixed-effect model for each outcome (mild or severe HL) was then built using a manual, stepwise backward variable-selection procedure, including the variables that were significant (*P* ≤ 0.05) as main effects in the sub-models. Variables associated (*P* ≤ 0.05) with the dependent variable were kept in the final models. All variables with *P * ≤ 0.20 for mild or severe HL in the univariable analyses were then re-tested one at a time in their respective final model, and kept in the model if they were significantly associated with the dependent variable. All plausible two-way interactions between the significant main effects were tested in the final models. Model fit was assessed by visual examination of residual plots [27, 28].

### Associations between HL and mastitis and culling

We also investigated possible associations between HL and mastitis incidence and culling with univariable mixed-effect logistic regression or mixed-effect linear regression analyses. Mild or severe HL was used as independent variable and the three dependent variables were: (1) milk SCC from test milking within 34 days before or after the visit; (2) veterinary-treated clinical mastitis (VTCM) within 90 days before or after the visit; and (3) culling of the cow within 90 days post-visit. Herd was included as random factor in all models. Parity, breed, DIM, milk yield, and visit period were added to the model if there was an association (*P* ≤ 0.05) between the dependent and the independent variable. This was to investigate them as confounding effects in the multivariable mixed-effect logistic model. Collinearity between variables was tested for all multivariable models, by calculations of Spearman rank correlations. Model fit for all models was assessed by visual examination of residual plots [27, 28].

## Results

### Herd and cow characteristics

In total, 3755 cows were examined (mean 38 cows/herd (SD 12.2); range 17–68 cows/herd), including 1901 Swedish Holstein (SH), 1300 Swedish Red (SR), 289 cross-breeds (SH × SR) and 265 cows of other breeds (mainly Jersey cows). There were few cows of other breeds in the herds, except for one herd with only Jersey cows; for this reason, cows of other breeds (n = 265) were excluded from the statistical analyses, including the Jersey herd. In addition, 273 cows were excluded because their hocks were too dirty for assessment of HL status. Thus, 3217 cows from 99 herds were included in the prevalence calculations and available for the statistical analyses. The mean annual herd size was 106 cows (SD 41.6; range 49–223 cows) and the mean annual milk production 9914 kg ECM/cow-year (SD 1236 kg; range–13,225 kg). Mean annual herd SCC was 247,000 cells/ml (SD 78,000; range 103,000–523,000 cells/ml).

### Prevalence of mild and severe HL

**Fig. 1:**
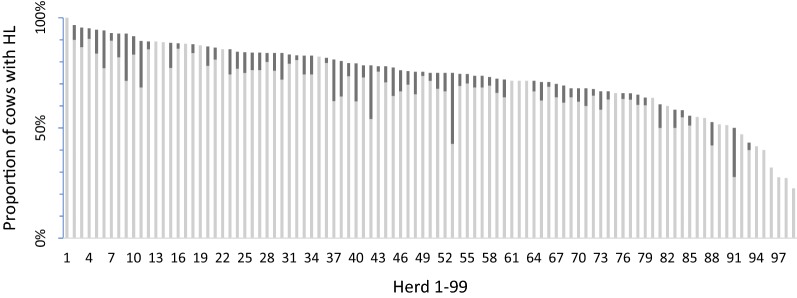
Prevalence (%) of mild (light grey) and severe (dark grey) hock lesions (HL) in 99 Swedish dairy herds sorted from highest to lowest HL prevalence (n = 3217 cows)

The overall cow prevalence of HL was 74% (2388 of 3217 cows; CI 73–76%), divided in 68% (2182 of 3217 cows; CI 66–69%) for mild HL and 6% (206 of 3217 cows; CI 6–7%) for severe HL. The within-herd prevalence of HL (Fig. 1) varied among the herds between 23 and 100% (mean 73%, SD 16%). For mild HL, the within-herd prevalence varied between 23 and 100% (mean 67%, SD 15%), and for severe HL, it varied between 0 and 32% (mean 6%, SD 6%). The herd prevalence for mild lesions was 100% (99/99 herds) and for severe lesions 76% (75/99 herds).

### Risk factors associated with mild HL

In total, 3009 cows from 99 herds were included in the univariable analyses, excluding cows with severe lesions. Of the 81 tested variables, 35 had *P* ≤ 0.20 in the univariable analysis. Thirty-two of these 35 variables were investigated in the 4 multivariable sub-models (6 in the cow-related, 11 in the general herd- and housing-related, 7 in the management-related and 8 in the herd health-related; Additional files 1 and 2). Three variables were excluded because of too many missing values (stocking ratio and farmer-reported presence of digital dermatitis in the herd), or too few cows in a specific category (reproduction disease within 90 days of visit). Independent variables associated with mild HL in the different sub-models (*P *≤ 0.05, Table 2) were then offered to the final model for mild HL. There were initially 8 variables in this final model of which 6 remained significant. After re-testing variables with *P *≤ 0.20 in the univariable analyses, bedding material was significant and included in the model. However, the association with production system (organic or conventional) was no longer significant (*P* = 0.076) and therefore removed. The total number of cows in the final model was 2447 from 79 herds.

**Table 2:** Cow- and herd-related variables associated with mild hock lesions (HL) in four multivariable mixed-effect logistic regression sub-models

Sub-model/variable	β	SE	OR	CI	*P*-value
Sub-model 1: cow-related factors (n = 2685)					
*Breed*					
Swedish Holstein (SH)	Ref				
Swedish Red (SR)	− 0.34	0.12	0.71	0.56–0.90	0.005
SH × SR	− 0.28	0.19	0.75	0.52–1.09	0.136
*DIM* (days)					
0–90	Ref				
91–180	0.27	0.12	1.31	1.04–1.65	0.022
180–305	0.62	0.12	1.87	1.46–2.38	< 0.001
> 305	0.24	0.16	1.28	0.93–1.74	0.127
Sub-model 2: general herd and housing-related factors (n = 2545 cows)					
*Cubicle base*					
Rubber mats	Ref				
Mattress	− 0.43	0.15	0.65	0.48–0.87	0.004
*Production system*					
Conventional	Ref				
Organic	− 0.47	0.19	0.62	0.43–0.91	0.014
Sub-model 3: management-related factors (n = 2685 cows)					
*Cow diet include maize silage*					
No	Ref				
Yes	0.40	0.20	1.50	1.01–2.21	0.042
*Type of feeding system*					
Automatic feeding stations for individual concentrate rations	Ref				
Total mixed rations (TMR)	− 0.54	0.23	0.58	0.37–0.90	0.016
Combination of TMR and individual concentration rations	− 0.11	0.17	0.90	0.64–1.26	0.535
*Type of milking parlor*					
Herringbone	Ref				
Tandem	− 0.47	0.15	0.62	0.46–0.84	0.002
Sub-model 4: herd health-related factors (n = 2587 cows)					
*Heifers > 17* *months not inseminated (%)*^a^					
0–16	Ref				
≥ 17	− 0.45	0.14	0.64	0.48–0.85	0.002

*β* regression coefficients, *OR* odds ratio, *CI* 95% confidence interval, *Ref* reference level

^a^Based on data from the Swedish Official Milk Recording Scheme for the 12 months preceding the herd visit

The final multivariable mixed-effect logistic regression model is presented in Table 3. Breed and DIM were significant cow-related risk factors. The risk for mild HL was lower in SR than in SH cows (OR 0.74; CI 0.59–0.94), whereas SH × SR cows had no difference in risk compared to either SH or SR cows. The risk for mild HL increased with higher DIM up to 305 DIM compared to 0–90 DIM (OR 1.35 and 2.06 for 91–180 days and 181–305 days respectively). This risk was higher for cows within 181–305 DIM than for cows in all other categories. Cows with more than 305 DIM did not differ significantly from cows within 0–90 or 91–180 DIM. Cows housed with mattresses as cubicle base had lower risk for mild HL than cows housed on rubber mats (OR 0.66). There was also a higher risk for cows housed on other bedding material categories compared to peat [sawdust (OR 2.39; CI 1.38–4.12), straw (OR 1.88; CI 1.04–3.4), or combinations of different bedding materials (OR 2.6; CI 1.25–5.39)]. The other categories of bedding material did not differ between each other. In addition, cows in herds with tandem milking parlors had a lower risk of mild HL than those in herringbone milking parlors (OR 0.75). Cows in herds with a high proportion of not yet inseminated heifers older than 17 months had a lower risk of mild HL than the herds with a low proportion (OR 0.61). There were no significant two-way interactions in this model.

**Table 3:** Cow- and herd-related factors associated with mild hock lesions (HL) in the final multivariable mixed-effect logistic regression model including 2447 cows in 79 Swedish dairy herds

Variables and categories	β	SE	OR	CI	*P*-value
Intercept	− 0.18	0.33			0.579
*Breed*					
Swedish Holstein (SH)	Ref				
Swedish Red (SR)	− 0.29	0.12	0.74	0.59–0.94	0.012
SH × SR	− 0.24	0.20	0.78	0.53–1.15	0.215
*Days in milk (DIM)*					
0–90	Ref				
91–180	0.30	0.12	1.35	1.05–1.72	0.017
181–305	0.72	0.13	2.06	1.59–2.66	< 0.001
> 305	0.30	0.17	1.35	0.97–1.88	0.074
*Bedding material*					
Peat	Ref				
Sawdust	0.87	0.28	2.39	1.38–4.12	0.002
Straw	0.63	0.30	1.88	1.04–3.40	0.035
Combination	0.95	0.37	2.60	1.25–5.39	0.011
*Cubicle base*					
Rubber mat	Ref				
Mattress	− 0.42	0.13	0.66	0.51–0.84	0.001
*Heifers > 17* *months not inseminated (%)*^a^					
0–16	Ref				
≥ 17	− 0.49	0.13	0.61	0.48–0.79	< 0.001
*Type of milking parlor*					
Herringbone	Ref				
Tandem	− 0.28	0.13	0.75	0.59–0.97	0.027

*β* regression coefficients, *OR* odds ratio, *CI* 95% confidence interval, *Ref* reference level

^a^Based on data from herd performance indicators from the Swedish Official Milk Recording Scheme

### Risk factors associated with severe HL

The evaluation of risk factors for severe HL included 1035 cows from 98 herds in the univariable analyses. Cows with mild lesions were excluded, including one herd with a prevalence of 100% mild HL. Of 81 analyzed variables, 22 had a *P* ≤ 0.20 and were kept for further analyses in one of the four multivariable sub-models (6 in the sub-model for cow-related, 6 in the herd and housing-related, 3 in the management-related, and 5 in the herd health-related factors sub-model; Additional files 1 and 2). Two risk factors were excluded (stocking ratio and farmer-reported presence of digital dermatitis in the herd) because of too many missing values. All variables significantly associated (*P* ≤ 0.05) with severe HL in the sub-models are presented in Table 4. These were then entered as main effects into the final model that initially contained 10 independent variables of which 6 remained significant. Variables with *P *≤ 0.20 in the univariable analyses were re-tested and kept in the final model if they had *P *≤ 0.05. Breed, cow hygiene, and use of teat disinfectant or other spray/dip applied after milking were variables that became significant at re-testing. Registered hoof disorder within 90 days was not significant (*P* = 0.057) and therefore removed from the final model. The random effect of cows within herd was not significant (*P* = 0.12), hence, a multivariable logistic regression model was used for the analysis instead of the mixed-effect model, this had a small effect (< 5% change) on the coefficients of the included variables.

**Table 4:** Cow- and herd-related variables associated with severe hock lesions (HL) in four multivariable mixed-effect logistic regression sub-models

Sub-model/variable	β	SE	OR	CI	*P*-value
Sub-model 1: cow-level factors (n = 936)					
*Breed*					
Swedish Holstein (SH)	Ref				
Swedish Red (SR)	− 0.76	0.24	0.47	0.29–0.75	0.002
SH × SR	− 0.49	0.34	0.61	0.31–1.19	0.149
*DIM* (days)					
0–90	Ref				
91–180	0.53	0.25	1.31	1.05–2.76	0.031
180–305	0.88	0.24	1.87	1.49–3.88	< 0.001
> 305	0.11	0.34	1.11	0.58–2.15	0.745
*Parity*					
First parity	Ref				
Second parity	− 0.24	0.25	0.79	0.49–1.28	0.34
Third or higher parity	0.68	0.22	1.96	1.29–3.0	0.002
*Registered hoof disorder of any type (registered at hoof trimmings within 90* *days from herd visit)*					
No	Ref				
Yes	0.75	0.29	2.11	1.19–3.74	0.011
Hoof trimming records not available	0.24	0.29	1.28	0.72–2.27	0.408
Sub-model 2: general herd and housing-related factors (n = 880 cows)					
*Average milk production (kg ECM/cow and year)* ^a^					
< 9800	Ref				
≥ 9800	0.51	0.24	1.66	1.04–2.65	0.032
*Cubicle width*					
Below recommendation	Ref				
As recommended	− 0.80	0.24	0.45	0.28–0.73	0.001
*Production system*					
Conventional	Ref				
Organic	− 1.07	0.33	0.34	0.18–0.66	0.001
Sub-model 3: management-related factors (n = 936 cows)					
*Type of milking parlor*					
Herringbone	Ref				
Tandem	− 0.84	0.27	0.43	0.25–0.74	0.002
Sub-model 4: herd health-related factors (n = 897 cows)					
*Heifers > 17* *months not inseminated (%)*^a^					
0–16	Ref				
≥ 17	− 0.59	0.25	0.55	0.34–0.91	0.02
*Presence of ectoparasites in cows during the last year according to farm owner/staff*					
No	Ref				
Yes	0.73	0.25	2.01	1.26–3.43	0.004

*β* regression coefficients, *OR* odds ratio, *CI* 95% confidence interval, *Ref* reference level

^a^Based on data from the Swedish Official Milk Recording Scheme for the 12 months preceding the herd visit

The final model for factors associated with severe HL included 919 cows from 88 herds (Table 5). Cow-related risk factors for severe lesions included breed and DIM. There was a lower risk for SR than SH cows (OR 0.6; CI 0.41–0.87). The risk increased with higher DIM (OR 1.59; CI 1.01–2.51) and OR 2.47 (CI 1.57–3.91) for 91–180 days and 181–305 days respectively when compared to 0–90 days), except for cows with > 305 DIM; for these cows there was no difference in risk compared to cows with 0–90 DIM. In addition, cows with hygiene score 4 (i.e., dirtier cows) had a lower risk of severe HL than cows with hygiene score 1–2 (OR 0.42; CI 0.23–0.79), and third or higher parity were associated with a higher risk of severe HL compared to first parity (OR 2.16; CI 1.45–3.23). Risk for severe HL was lower in cows milked in tandem milking parlors than in cows milked in herringbone ones (OR 0.47; CI 0.31–0.69). Furthermore, cows in herds with recommended cubicle widths had lower risk (OR 0.36; CI 0.24–0.52) than cows in smaller cubicles. The risk was also lower for cows in herds that used teat dip (OR 0.47; CI 0.26–0.85) or no treatment (OR 0.32; CI 0.12–0.87) after milking than those that used teat spray. Finally, the variable with the largest effect size was that cows in organic herds had a lower risk (OR 0.21; CI 0.13–0.36) than those in conventional herds. There were no significant two-way interactions in this model. The model explained about 15% of the variation in the data (R^2^ = 0.145).

**Table 5:** Cow- and herd-related factors associated with severe hock lesions (HL) in the final multivariable logistic regression model including 919 cows from 88 Swedish dairy herds

Variables and categories	β	SE	OR	CI	*P*-value
Intercept	− 0.96	0.37			0.011
*Breed*					
Swedish Holstein (SH)	Ref				
Swedish Red (SR)	− 0.52	0.19	0.60	0.41–0.87	0.008
SH × SR	− 0.16	0.31	0.78	0.46–1.55	0.596
*Days in milk*					
0–90	Ref				
91–180	0.46	0.23	1.59	1.01–2.51	0.047
181–305	0.91	0.23	2.47	1.57–3.91	< 0.001
> 305	0.11	0.31	1.12	0.60–2.07	0.723
*Hygiene score* ^a^					
1–2	Ref				
3	− 0.17	0.21	0.84	0.56–1.26	0.404
4	− 0.86	0.32	0.42	0.23–0.79	0.007
*Parity*					
First parity	Ref				
Second parity	− 0.11	0.23	0.90	0.57–1.42	0.638
Third or higher parity	0.77	0.21	2.16	1.45–3.23	< 0.001
*Cubicle width*					
Below recommendation	Ref				
As recommended	− 1.03	0.19	0.36	0.24–0.52	< 0.001
*Production system*					
Conventional	Ref				
Organic	− 1.54	0.27	0.21	0.13–0.36	< 0.001
*Teat disinfectant or other spray/dip applied after milking*					
Spray	Ref				
Dip	− 0.75	0.30	0.47	0.26–0.85	0.012
None	− 1.15	0.52	0.32	0.12–0.87	0.026
*Type of milking parlor*					
Herringbone	Ref				
Tandem	− 0.76	0.20	0.47	0.31–0.69	< 0.001

*β* regression coefficients, *OR* odds ratio, *CI* 95% confidence interval, *Ref* reference level

^a^Hygiene score 1–4: (1) completely clean, (2) manure stains on hind limb and/or udder (1 and 2 merged due to few observations in category 1), (3) one to two areas of manure patches of at least 10 cm in diameter, and (4) more than 2 areas of manure patches as described for score 3

### Associations between hock lesions and mastitis and culling

The analyses of associations between mild HL and VTCM within 90 days before or after herd visit, or culling within 90 days post-visit, included 2911 cows from 97 herds. The analyses of associations between mild HL and milk SCC included 2884 cows from 97 herds. In the univariable mixed-effect linear regression analysis, mild HL showed no significant association with any of the outcomes (*P* > 0.05).

For severe HL, 996 cows from 96 herds were included in the analyses of association with VTCM within 90 days before or after herd visit, and culling within 90 days post-visit. The analysis of associations between severe HL and milk SCC included 988 cows from 96 herds. An association between severe HL and culling was seen in the univariable analysis (*P* = 0.009), but the association was no longer significant (*P* = 0.11) when parity, milk yield and DIM were included. There were no significant associations between severe HL and VTCM or milk SCC.

## Discussion

This study is the first large-scale investigation on the prevalence of, and risk factors for HL in Swedish freestall dairy herds, and it contributes to better understanding of HL epidemiology in Sweden.

Hock lesions are a common problem in Swedish freestalls, affecting 74% of the cows in our study. Moreover, the true prevalence could be even higher as only the outside of one hock per cow was examined. However, a previous large-scale study on HL found that most HL were bilateral, and that medial lesions rarely occurred if the cow had no lateral lesion on the same leg [8]. Similarly, the risk for HL did not differ depending on which hock (right or left) that was examined in the present study (data not shown). Logically, if a cow spends an equal amount of time lying down on her left and right side, bilateral lesions are likely, as the external and internal conditions for both hocks are similar, at least for a non-lame cow. Our reported prevalence of HL is considerably higher than that in a previous Swedish study [20]. However, that study used a different definition of HL, and the majority of included herds had tie-stalls. Our observed prevalence (68%) of mild HL (hair loss) is in line with results from studies in freestalls in other countries (ranging from 50% [3, 6] to 82% [8, 12]). We found an overall prevalence of 6% for severe HL (ulceration and/or evident swelling). Similar results have been reported from Norway [3] and northeastern USA [12], whereas other studies have reported higher prevalence of severe manifestations of HL (up to 36%) [8, 9]. Differences between scoring systems and observer subjectivity make comparisons between studies difficult. The problems associated with the lack of a common scoring system have been highlighted in several studies [8, 29, 30]. The hock assessment chart for cattle [25] in our study is easy to use in field studies and allows fast and accurate scoring of HL. However, registration of hair loss, swellings and ulcerations as separate, rather than combined, events would probably yield more information on the etiology of such lesions. In line with this, Potterton et al. [16] found that risk factors differ depending on the HL manifestation. As there are pros and cons with the current scoring systems, care must be taken in selection of the method, interpretation of the results and comparison with other studies. It is also important that the methods used are reported accurately to enable comparisons and reproducibility of studies.

Even though many herds had a high HL prevalence, the between-herd variation was large, especially for severe HL (0–32%). This variation, also seen by others [3, 8, 14], indicates that herd and management factors affect the HL occurrence. Our study identified several risk factors, especially cow-related ones, that were common to both mild and severe HL; however other risk factors were specific to the type of HL. The discussion below on risk factors for HL is based on the design of the sub-models and divided into cow-, general herd and housing-, management and herd health-related risk factors.

### Cow-related risk factors

Breed and DIM were associated with both mild and severe HL in a similar way. The SH cows had a greater risk for HL than the SR cows. A higher risk for Holstein has also been seen in other studies compared to Jerseys and local breeds (e.g., Danish Red) [16, 31]. Generally, SH cows are larger and have a lower body condition score (BCS) than SR cows [32, 33]. Large cows (measured by hip width) [34] and low BCS [3] have an increased risk for HL, which could partly explain the difference in risk between SH and other breeds.

An increased risk for HL with increasing DIM is in line with other studies on all HL [3], severe HL [10], and swelling at the hock [16]. It is likely that a lesion that develops in early lactation persists throughout the lactation period. An interesting, previously unreported, observation was that cows with more than 305 DIM (i.e. a longer than average lactation period) had a lower risk of HL than cows within 91–180 and 181–305 DIM. This might be because there were fewer cows in this category, but it might also indicate that HL improve with time, perhaps due to repaired body condition. The association between HL and DIM needs further investigation.

Higher parity and lower hygiene scores (i.e., cleaner cows) were significant risk factors for severe HL, but not for mild HL. Other studies have shown the same association between parity and mild [16] or all HL [3, 17]. In our study, however, mild HL was very common in all parities. Higher parity is associated with a higher risk for other conditions, including most types of hoof lesions [35], lameness [36] and mastitis [37], which may indicate a reduced ability of older cows to cope with injuries and diseases. As indications of a causal relationship between lameness and HL was found by Lim et al. [30], the increased risk for lameness in older cows might also increase the risk of severe HL. In addition, older cows may be less physically agile, and therefore contract more injuries when rising and lying [17]. The lower risk for severe HL in the cows that were most dirty on the hind leg above the hock and on the side of the udder is in line with Potterton et al. [16]. It could be speculated that a layer of dirt may protect the skin integrity, or that certain management or housing factors, such as amount of bedding material in cubicles, affect both cow hygiene and severe HL. It may also be harder to detect HL on a dirty hock, but this should not be a major bias in our study because we excluded the cows that were too dirty for assessment of hock status.

Several studies have found associations between HL and lameness [3, 16, 17, 31]. In our study, we saw a significant association between registration of at least one hoof disorder within 90 days before or after HL registration and increased risk of severe HL. This association was seen in the univariable analysis and in the cow-related multivariable sub-model, but not in the final model. Hoof trimming records were, however, only available for 46 of the herds, which could have affected the results of the final model.

### General herd and housing-related risk factors

We found no common housing-related risk factors for mild and severe HL, which is in line with Potterton et al. [16]. Cubicle base and bedding material were associated with mild HL. Mattress and peat were associated with a lower risk than rubber mats and other types of bedding material, respectively. Mattresses are usually thicker and softer than rubber mats which leads to less pressure on the hock when the cow is lying down, and thus, less risk of impaired circulation and subsequent tissue damage. This finding is supported by previous studies investigating different cubicle bases and their softness [3, 9, 23]. However, compared to deep straw or sand bedding, mattresses and rubber mats have been associated with a higher risk of HL [6, 13, 38]. The effect of bedding material on HL has also been investigated in several studies, and sand is usually associated with a lower risk for HL than traditional bedding materials such as sawdust and straw [16, 22]. However, the combination of cubicle base, and type and depth of bedding material, probably has a large effect on the risk for HL, as these all affect the conditions of the lying surface [13, 22]. In our study, there were too few (5) herds with deep straw or sand bedding to evaluate their effects on HL prevalence. Changing bedding from straw to peat reduced the severity of HL in a Swedish study [39], but we found no studies from other countries where peat was compared with other bedding materials in relation to HL.

Cows in herds with organic production had a notably lower risk for severe HL than cows in conventional herds. The trend was similar for mild HL, but not statistically significant in the final model. This finding is in line with other studies [3, 17]. Swedish regulations for dairy cow housing and management are similar in conventional and organic production, but the regulations for e.g., feed concentrate intake and access to pasture, are stricter in organic herds [40]. Cows in organic herds generally spend more time on pasture as they are required to have access to pasture for a longer time each day (12 h compared to 6 h) during the legislated pasture period (2–4 months/year depending on geographical location), and to have more of their nutritional needs fulfilled via pasture than cows in conventional herds [40, 41]. The organic herds also have part-time outdoor access for two additional months/year [40]. More time on pasture (both total length and hours/day) has been associated with reduced prevalence of HL and lameness [17, 31, 42]. In the present study, we investigated the length (days on pasture) and extent (if cows were outside for at least 12 h/day or not) of the most recent pasture period, but found no significant associations with HL. However, cows in organic herds may have other benefits from the pasture period that reduces their risk for severe HL compared to conventional herds. There may also be other, unidentified, management factors that differ between organic and conventional herds that affect the occurrence of severe HL.

The only housing-related factor associated with severe HL was cubicle width. Wider cubicles (i.e., of recommended dimensions compared to those under the recommendations) reduced the risk for severe HL, which is likely due to more space for the cow to lie down or stand up. To our knowledge, this association has not been reported previously, but several other cubicle dimensions have been associated with increased risk for HL. These include cubicle length [3, 9, 16], total standing area per cow [17], neck rail distance from curb [10, 16], height of neck rail [16] and height of lowest horizontal rail of cubicle partition [9, 16]. We had expected more cubicle dimensions to be associated with HL also in the present study, but individual housing risk factors can be difficult to identify due to complex interactions between cubicle design and type of bedding, cow factors, and management routines. In addition, most herds were in the “under recommendation”-category for some measurements (e.g. neck rail height and distance from rear curb), which makes possible effects of having correctly dimensioned cubicles hard to evaluate.

### Management-related risk factors

Cows milked in tandem parlors had a lower risk of both mild and severe HL than cows milked in herringbone ones. To our knowledge this association has not been previously investigated. We speculate that the risk for traumatic skin injuries (due to crowding and physical conflicts with housing elements) may be greater in the herringbone milking parlors. In these parlors, several cows (generally 6–12 under Swedish conditions) move in and out at the same time, whereas they move one at a time in the tandem parlors. However, unidentified herd or management factors may also differ between herds in the two types of milking parlors. The reasons for the finding that use of teat spray after milking was associated with a higher risk of severe HL than use of teat dip or no dip/spray are not known. The association may reflect other management or herd factors not analyzed in this study or it could be a spurious finding due to the large number of independent variables that were analyzed.

### Herd health-related risk factors

The only significant association between HL and herd health-related factors in the final models was an increased risk for mild HL in herds with a low proportion of not yet inseminated heifers > 17 months. The same association was seen for severe HL in the herd health-related sub-model, but not in the final analysis. Similar results were obtained by Rutherford et al. [17], who discussed that their results could have been related to organic farming rather than not yet insemination per se, as organic herds mated heifers later than conventional herds. Among our included herds, 43% of the conventional herds compared to 70% of the organic herds had a high proportion of not yet inseminated heifers (data not shown), indicating that this might be true also in our study and that these associations require further investigations.

### Associations between HL and udder health and culling

We found no significant associations between mild or severe HL and udder health. Overall, there were few cows with registered VTCM in the SOMRS within 90 days before or after the herd visit. However, diseases are underreported to the SOMRS database [43], which could have affected our results. Hock lesions have been associated with increased risk of clinical mastitis [18] and high herd-level SCC in freestalls [6], but our results showed no such associations. Results from a previous Swedish study indicate a role of HL in transmission of the common udder pathogen *Staphylococcus aureus* in dairy herds [44]. Thus, associations between HL and udder health should be further investigated in longitudinal studies.

The univariable analysis showed that cows with severe HL had an increased risk for culling within 90 days post-visit, but when the combined effects of parity, milk yield and DIM were included in the model, this association was not significant. However, culling decisions are often based on a combination of factors, and severe HL could contribute to such a decision for affected cows.

### Additional comments

The present cross-sectional study was designed to investigate several potential cow- and herd-related risk factors in a large number of Swedish dairy herds. However, a cross-sectional study only yields a snapshot of the conditions in the herds. Moreover, the large number of investigated independent variables increases the risk of type I errors (i.e. identification of risk factors that are not true risk factors). As we use a mixed effect model, that takes into account the random effect of herd, the point estimates and their intervals move toward each other. This makes the comparisons more conservative and reduces the risk for such errors. Still, some of the findings are probably due to chance and this kind of study need to be repeated to confirm true risk factors. Moreover, when the number of observations is large, small differences in the estimates might become significant although the biological effect is not of importance on population level. There is also a risk for selection bias in this type of study, as herds with HL problems might be more inclined to participate. However, the herd characteristics and inclusion process did not indicate that this was the case for our study, as most contacted herds were willing to participate, and the study also investigated udder cleft dermatitis lesions.

As previously stated, the complexity of dairy production makes it difficult to identify individual variables affecting the prevalence of HL. In line with this, most significant variables found in our study had relatively low effect size. Moreover, the final model for severe HL only explained 15% of the variation in the dataset. Thus, there might be other factors of importance that we did not register in the present study. In our study we combined cow- and herd-related factors in the statistical analyses, which could make it difficult to find significant herd-related factors as the cow-related factors seem to be more closely associated with HL. Moreover, farmers’ attitudes and awareness of HL may influence management routines and HL occurrence. Benchmarking of cow comfort in dairy herds in Canada and the USA reduced HL prevalence in 14 dairy herds, because the farmers became more aware of the lesions and addressed the issue, mainly by improving cubicle bedding [45]. Mild HL is such a common finding in dairy herds that farmers might not notice them or consider them a problem [8]. However, mild HL have been associated with lameness [16] and may also contain udder pathogens [44], and should not be overlooked as a welfare problem in dairy herds.

## Conclusions

HL is a common finding in Swedish freestall dairy herds. Hair loss (i.e., mild HL) was observed on cows in all herds, with more than 50% of cows affected in 88 of the 99 herds. The overall prevalence of severe lesions was 6%, but markedly higher within-herd prevalence was seen in some herds (up to 32%). Several cow- and herd-related risk factors were identified. Some were common to both mild and severe lesions, such as breed, DIM and type of milking parlor. Other risk factors were associated only with mild lesions, (e.g., cubicle base and bedding material), or severe lesions, (e.g., production system and cubicle width). Our results, combined with those of previous studies on HL, indicate that there are ways to reduce HL prevalence in Swedish dairy herds. Preventive measures may include providing a soft lying area for the cows, such as mattresses or deep bedding. Using peat as bedding material and providing cubicles with appropriate width could also reduce the risk of HL. Longitudinal studies of HL are warranted to further increase the understanding of the etiology and pathogenesis of these lesions.

## Additional files

**Additional file 1.** Results of univariable analyses of cow-related risk factors analyzed in mixed-effect logistic regression models for their association with mild and severe hock lesions (HL), based on observations of 3217 cows in 99 Swedish dairy herds, including how the information was obtained and the number of cows in each category.

**Additional file 2.** Results of univariable analyses of 3 sub-groups of herd-related risk factors (general herd and housing-related, management-related and health-related factors) analyzed in mixed-effect logistic regression models for their association with mild and severe hock lesions (HL) based on observations of 3479 cows in 99 Swedish dairy herds. The table also contains information on how the information was obtained and the number of cows in each category.
