# Supplementary material for: Hock lesions in dairy cows in freestall herds: a cross-sectional study of prevalence and risk factors
Source: Acta Vet Scand. 2018 Aug 13;60:47. doi: 10.1186/s13028-018-0401-9 (PMC6090646; doi:10.1186/s13028-018-0401-9)
Supplement: Supplementary file 1 — Additional file 1. Results of univariable analyses of cow-related risk factors analyzed in mixed-effect logistic regression models for their association with mild and severe hock lesions (HL), based on observations of 3217 cows in 99 Swedish dairy herds, including how the information was obtained and the number of cows in each category. [file 13028_2018_401_MOESM1_ESM.docx]

**Additional file 1.** Results of univariable analyses of cow-related risk factors analyzed in mixed-effect logistic regression models for their association with mild and severe hock lesions (HL), based on observations of 3,217 cows in 99 Swedish dairy herds

| Variables and categories^1^ | Number of cows with no, mild or severe HL | | | I^2^ | *P-*value | |
| --- | --- | --- | --- | --- | --- | --- |
|  | No  HL | Mild  HL | Severe HL |  | Mild HL | Severe HL |
| Breed |  |  |  | C | 0.002 | 0.014 |
| Swedish Holstein (SH) | 402 | 1,234 | 124 |  |  |  |
| Swedish Red (SR) | 347 | 781 | 62 |  |  |  |
| Cross-breed, SH x SR | 80 | 167 | 20 |  |  |  |
| Days in milk |  |  |  | C | < 0.001 | 0.001 |
| 0 - 90 | 284 | 573 | 47 |  |  |  |
| 91 - 180 | 257 | 640 | 63 |  |  |  |
| 181 - 305 | 185 | 697 | 74 |  |  |  |
| > 305 | 103 | 272 | 22 |  |  |  |
| Hygiene score |  |  |  | O | 0.127 | 0.034 |
| 1^3^ | 12 | 43 | 7 |  |  |  |
| 2^3^ | 482 | 1,329 | 133 |  |  |  |
| 3 | 214 | 533 | 51 |  |  |  |
| 4 | 121 | 276 | 15 |  |  |  |
| Parity |  |  |  | C | 0.019 | < 0.001 |
| 1 | 326 | 774 | 64 |  |  |  |
| 2 | 253 | 639 | 47 |  |  |  |
| ≥ 3 | 250 | 769 | 95 |  |  |  |
| Presence of udder cleft dermatitis |  |  |  | O | 0.035 | 0.45 |
| No | 601 | 1,482 | 151 |  |  |  |
| Yes | 225 | 696 | 55 |  |  |  |
| Milk production (kg ECM/day)^4^ | | | | C | 0.53 | 0.40 |
| < 27 | 195 | 510 | 46 |  |  |  |
| 27 - 31 | 194 | 493 | 45 |  |  |  |
| 32 - 37 | 194 | 563 | 59 |  |  |  |
| ≥ 38 | 207 | 544 | 52 |  |  |  |
| Milk SCC (cells/ml)^4^ | | | | C | 0.48 | 0.141 |
| < 32,000 | 209 | 516 | 39 |  |  |  |
| 32,000 – 70,999 | 191 | 526 | 62 |  |  |  |
| 71,000 – 191,999 | 197 | 538 | 44 |  |  |  |
| ≥ 192,000 | 189 | 520 | 57 |  |  |  |
| Milk urea concentration? (mM/l)^4^ |  |  |  | C | 0.50 | 0.64 |
| < 3.8 | 205 | 486 | 41 |  |  |  |
| 3.8 - 4.3 | 181 | 484 | 46 |  |  |  |
| 4.3 - 5.1 | 217 | 581 | 52 |  |  |  |
| ≥ 5.2 | 183 | 549 | 63 |  |  |  |
| Registered hoof disorder of any type^5^ | | | | C | 0.174 | < 0.001 |
| No | 200 | 509 | 40 |  |  |  |
| Yes | 175 | 500 | 62 |  |  |  |
| Records not available | 454 | 1,173 | 104 |  |  |  |
| Registered hoof eczema^5,6^ | | | | C | 0.45 | 0.81 |
| No | 324 | 886 | 86 |  |  |  |
| Yes | 51 | 123 | 16 |  |  |  |
| Records not available | 454 | 1,173 | 104 |  |  |  |
| Clinical mastitis^7^ |  |  |  | C | 0.35 | 0.67 |
| No | 794 | 2,069 | 200 |  |  |  |
| Yes | 35 | 113 | 6 |  |  |  |
| Hoof- or leg disease^7^ |  |  |  | C | 0.30 | 0.92 |
| No | 821 | 2,171 | 204 |  |  |  |
| Yes | 8 | 11 | 2 |  |  |  |
| Metabolic disease^7^ |  |  |  | C | 0.90 | 0.95 |
| No | 813 | 2,139 | 203 |  |  |  |
| Yes | 16 | 43 | 3 |  |  |  |
| Reproduction disease^7^ |  |  |  | C | 0.14 | 0.86 |
| No | 821 | 2,170 | 204 |  |  |  |
| Yes | 8 | 12 | 2 |  |  |  |
| Treatment due to any disease, including the above^7^ | | | | C | 0.69 | 0.33 |
| No | 752 | 1,970 | 187 |  |  |  |
| Yes | 77 | 212 | 19 |  |  |  |
| Culled within 90 days after visit | | | | C | 0.27 | 0.009 |
| No | 780 | 2,026 | 183 |  |  |  |
| Yes | 49 | 156 | 23 |  |  |  |

^1^ Continuous variables were assessed if they were linearly related to the outcome, and if not, they were categorized using percentiles as cut-offs (except for DIM that was categorized into early (0 - 90 days), mid- (91 - 180 days) and late (181 - 305 days) lactation as this represents an average lactation period and cows with ≥ 306 days in a fourth category as those lactations were considered longer than normal

^2^ I = Information obtained via: O = observed at herd visit, Q = questionnaire, answers obtained via interviewing farm owner or staff, C = data from the Swedish Official Milk Recording Scheme (SOMRS)

^3^ Due to few observations with hygiene score 1, category 1 and 2 were merged in the analyses.

^4^ Results from test milking within 34 days before or after visit, data missing from 2 herds in the SOMRS

^5^ Registrations of hoof disorders from hoof trimmings within 90 days before or after visit
(data available from 53 of 99 herds)

^6^ Hoof eczema defined as skin dermatitis/skin infection or digital dermatitis

^7^ Registrations of veterinary treated diseases within 90 days before or after herd visit
