# Supplementary material for: Hock lesions in dairy cows in freestall herds: a cross-sectional study of prevalence and risk factors
Source: Acta Vet Scand. 2018 Aug 13;60:47. doi: 10.1186/s13028-018-0401-9 (PMC6090646; doi:10.1186/s13028-018-0401-9)
Supplement: Supplementary file 2 — Additional file 2. Results of univariable analyses of 3 sub-groups of herd-related risk factors (general herd and housing-related, management-related and health-related factors) analyzed in mixed-effect logistic regression models for their association with mild and severe hock lesions (HL) based on observations of 3479 cows in 99 Swedish dairy herds. The table also contains information on how the information was obtained and the number of cows in each category. [file 13028_2018_401_MOESM2_ESM.docx]

**Additional file 2.** Results of univariable analyses of 3 sub-groups of herd-related risk factors (general herd and housing-related, management-related and health-related factors) analyzed in mixed-effect logistic regression models for their association with mild and severe hock lesions (HL) based on observations of 3,479 cows in 99 Swedish dairy herds

| Variables and categories^1^ | Number of cows with no, mild or severe HL^2^ | | | I^3^ | Results of univariable analysis  *(P*-value) | |
| --- | --- | --- | --- | --- | --- | --- |
|  | No  HL | Mild HL | Severe HL |  | Mild  HL | Severe HL |
| **General herd and housing-related factors** | | | |  |  |  |
| Herd size |  |  |  | C | 0.95 | 0.58 |
| < 75 | 146 | 355 | 28 |  |  |  |
| 75 – 98 | 194 | 534 | 41 |  |  |  |
| 99 – 125 | 230 | 590 | 63 |  |  |  |
| ≥ 126 | 259 | 703 | 74 |  |  |  |
| Production system |  |  |  | C | 0.018 | 0.008 |
| Conventional | 644 | 1,813 | 183 |  |  |  |
| Organic | 185 | 369 | 23 |  |  |  |
| Average milk production (kg ECM/cow and year)^4^ | | | | C | 0.067 | 0.005 |
| < 9,800 | 401 | 943 | 68 |  |  |  |
| ≥ 9,800 | 392 | 1,177 | 135 |  |  |  |
| Average slaughter weight (kg) |  |  |  | C | 0.66 | 0.31 |
| < 282 | 150 | 412 | 40 |  |  |  |
| 282 - 295 | 195 | 536 | 50 |  |  |  |
| 295 - 307 | 134 | 452 | 50 |  |  |  |
| ≥ 308 | 211 | 506 | 40 |  |  |  |
| Type of stable |  |  |  | Q | 0.037 | 0.30 |
| Not insulated | 233 | 517 | 50 |  |  |  |
| Insulated | 582 | 1,627 | 153 |  |  |  |
| Year cubicles were installed |  |  |  | Q | 0.032 | 0.26 |
| < 2001 | 276 | 921 | 80 |  |  |  |
| 2001 - 2005 | 197 | 524 | 59 |  |  |  |
| ≥ 2006 | 320 | 644 | 60 |  |  |  |
| Stocking ratio (cows/cubicles)^5^ |  |  |  | Q | 0.053 | 0.17 |
| < 1 | 93 | 303 | 23 |  |  |  |
| 1 | 237 | 622 | 51 |  |  |  |
| > 1 | 126 | 569 | 52 |  |  |  |
| Type of alley flooring |  |  |  | O | 0.19 | 0.59 |
| Concrete or rubber with manure scrapers | 526 | 1,318 | 125 |  |  |  |
| Slatted floor | 303 | 864 | 81 |  |  |  |
| Type of cubicles |  |  |  | O | 0.25 | 0.066 |
| Conventional lying cubicles | 750 | 1,945 | 178 |  |  |  |
| Combination of eating and lying cubicles | 56 | 202 | 26 |  |  |  |
| Cubicle base |  |  |  | O | < 0.001 | < 0.001 |
| Rubber mats | 327 | 1,117 | 122 |  |  |  |
| Rubber mattress | 386 | 871 | 72 |  |  |  |
| Combination^6^ | 36 | 104 | 10 |  |  |  |
| Concrete^6^ | 42 | 34 | 2 |  |  |  |
| Deep straw bedding^6^ | 38 | 56 | 0 |  |  |  |
| Concrete exposed behind mat/mattress at rear curb | | | | O | 0.01 | 0.014 |
| No | 298 | 886 | 81 |  |  |  |
| Yes | 434 | 1,198 | 123 |  |  |  |
| No mat/mattress | 80 | 90 | 2 |  |  |  |
| Bedding material |  |  |  | O | 0.068 | 0.22 |
| Wood shavings | 552 | 1,603 | 147 |  |  |  |
| Straw | 146 | 324 | 25 |  |  |  |
| Peat | 62 | 91 | 18 |  |  |  |
| Combination | 59 | 147 | 16 |  |  |  |
| None^6^ | 10 | 17 | 0 |  |  |  |
| Cubicle length |  |  |  | O | 0.46 | 0.67 |
| Below recommendation | 530 | 1,509 | 139 |  |  |  |
| As recommended (275 ± 2 cm) | 40 | 118 | 12 |  |  |  |
| Above recommendation | 220 | 500 | 46 |  |  |  |
| Cubicle width |  |  |  | O | 0.056 | 0.017 |
| Below recommendation | 443 | 1,387 | 147 |  |  |  |
| As recommended (125 ± 2 cm) | 355 | 760 | 59 |  |  |  |
| Above recommendation | 0 | 0 | 0 |  |  |  |
| Distance from brisket board to rear curb  (or total length of cubicle if no brisket board) | | | | O | 0.51 | 0.43 |
| Below recommendation | 475 | 1,175 | 107 |  |  |  |
| As recommended (180 ± 2 cm) | 175 | 516 | 48 |  |  |  |
| Above recommendation | 162 | 450 | 51 |  |  |  |
| Distance from neck rail to rear curb |  |  |  | O | 0.47 | 0.42 |
| Below recommendation | 571 | 1,410 | 129 |  |  |  |
| As recommended (175 ± 5 cm) | 145 | 447 | 45 |  |  |  |
| Above recommendation | 16 | 56 | 5 |  |  |  |
| Neck rail height |  |  |  | O | 0.021 | 0.26 |
| Below recommendation | 607 | 1,715 | 159 |  |  |  |
| As recommended (125 ± 5 cm) | 108 | 186 | 19 |  |  |  |
| Above recommendation^7^ | 17 | 12 | 1 |  |  |  |
| Upper front rail height |  |  |  | O | 0.35 | 0.45 |
| Below recommendation | 330 | 830 | 65 |  |  |  |
| As recommended (90 ± 5 cm) | 207 | 483 | 36 |  |  |  |
| Above recommendation | 144 | 418 | 47 |  |  |  |
| Lower front rail height |  |  |  | O | 0.31 | 0.46 |
| Below recommendation | 0 | 0 | 0 |  |  |  |
| As recommended (10 ± 5 cm) | 11 | 22 | 1 |  |  |  |
| Above recommendation | 212 | 476 | 46 |  |  |  |
| Rear curb height |  |  |  | O | 0.89 | 0.51 |
| Below recommendation | 121 | 283 | 34 |  |  |  |
| As recommended (20 ± 2 cm) | 240 | 633 | 46 |  |  |  |
| Above recommendation | 439 | 1,191 | 123 |  |  |  |
| Bottom side rail height, measured 70 cm from rear curb | | | | O | 0.19 | 0.43 |
| As recommended (< 55 / > 70 ± 2 cm) | 431 | 1,059 | 97 |  |  |  |
| Not as recommended | 310 | 961 | 80 |  |  |  |
|  |  |  |  |  |  |  |
| **Management-related factors:** |  |  |  |  |  |  |
| Length of previous pasture period (days) | | | | Q | 0.77 | 0.63 |
| < 121 | 232 | 673 | 68 |  |  |  |
| 121 - 136 | 195 | 440 | 52 |  |  |  |
| 137 - 149 | 177 | 457 | 34 |  |  |  |
| ≥ 150 | 220 | 575 | 51 |  |  |  |
| Cows stayed out for more than 50% of the day during pasture period | | | | Q | 0.90 | 0.74 |
| No | 166 | 534 | 45 |  |  |  |
| Yes | 328 | 994 | 81 |  |  |  |
| Length of winter housing at visit (days) |  |  |  | Q | 0.88 | 0.36 |
| 100 - 126 | 199 | 490 | 51 |  |  |  |
| 127 - 152 | 175 | 516 | 65 |  |  |  |
| 153 - 172 | 195 | 555 | 46 |  |  |  |
| >172 | 260 | 621 | 44 |  |  |  |
| Frequency of cleaning by alley scrapers (times/day) | | | | Q | 0.135 | 0.041 |
| < 12 | 131 | 231 | 15 |  |  |  |
| 12 - 23 | 138 | 348 | 23 |  |  |  |
| ≥ 24 | 183 | 611 | 72 |  |  |  |
| Frequency of cleaning of cubicles (times/day) | | | | Q | 0.61 | 0.82 |
| < 2 | 13 | 25 | 2 |  |  |  |
| 2 | 381 | 1,195 | 100 |  |  |  |
| > 2 | 119 | 375 | 39 |  |  |  |
| Frequency of adding new bedding material (times/week) | | | | Q | 0.28 | 0.87 |
| < 1 | 98 | 357 | 30 |  |  |  |
| 1 - 6 | 384 | 852 | 92 |  |  |  |
| ≥ 7 | 325 | 935 | 84 |  |  |  |
| Frequency of hoof trimming (times/year) |  |  |  | Q | 0.036 | 0.65 |
| 1 | 170 | 389 | 43 |  |  |  |
| 2 | 516 | 1,450 | 126 |  |  |  |
| 3 | 92 | 293 | 31 |  |  |  |
| When needed^6^ | 51 | 50 | 6 |  |  |  |
| Type of milking parlor |  |  |  | O | 0.096 | 0.035 |
| Herringbone | 466 | 1,356 | 149 |  |  |  |
| Tandem | 349 | 812 | 54 |  |  |  |
| Cows milked in tie-stall^6^ | 14 | 14 | 3 |  |  |  |
| Size of milking parlor (number of cows) | | | | O | 0.018 | 0.008 |
| 3 x 2 | 79 | 336 | 28 |  |  |  |
| 4 x 2 | 240 | 441 | 26 |  |  |  |
| 5-6 x 2 | 138 | 350 | 30 |  |  |  |
| 7-8 x 2 | 242 | 756 | 77 |  |  |  |
| > 8 x 2 | 116 | 285 | 42 |  |  |  |
| Longest interval between two milkings (hours) | | | | Q | 0.71 | 0.94 |
| ≤ 10 (cows milked 3 times/day) | 75 | 185 | 22 |  |  |  |
| ≥ 12 (cows milked 2 times/day) | 754 | 1,997 | 184 |  |  |  |
| Rinsing of cows and/or milking organs when cows  were present in the milking parlor | | | | O | 0.43 | 0.87 |
| Never | 211 | 510 | 52 |  |  |  |
| Sometimes | 308 | 827 | 78 |  |  |  |
| Often | 303 | 825 | 75 |  |  |  |
| Premilking routine include use of water or spray on teats or udders | | | | O | 0.34 | 0.95 |
| No | 528 | 1,357 | 125 |  |  |  |
| Yes | 259 | 686 | 54 |  |  |  |
| Teat disinfectant or other spray/dip applied after milking | | | | O | 0.196 | 0.021 |
| Spray | 615 | 1,766 | 182 |  |  |  |
| Dip | 137 | 296 | 19 |  |  |  |
| None | 77 | 120 | 5 |  |  |  |
| Feeding system |  |  |  | Q | 0.085 | 0.74 |
| Automatic feeding stations for individual concentrate rations | 335 | 740 | 83 |  |  |  |
| TMR | 171 | 461 | 53 |  |  |  |
| Combination of TMR and individual concentration rations | 323 | 981 | 70 |  |  |  |
| Cows fed in milking parlor |  |  |  | O | 0.31 | 0.54 |
| No | 711 | 1,861 | 188 |  |  |  |
| Yes | 101 | 313 | 18 |  |  |  |
| Regular use of feed adviser |  |  |  | Q | 0.41 | 0.36 |
| No | 148 | 426 | 43 |  |  |  |
| Yes | 681 | 1,756 | 163 |  |  |  |
| Maize silage included in cow diet |  |  |  | Q | 0.192 | 0.033 |
| No | 695 | 1,737 | 148 |  |  |  |
| Yes | 134 | 445 | 58 |  |  |  |
| Wheat included in cow diet |  |  |  | Q | 0.57 | 0.57 |
| No | 466 | 1,311 | 123 |  |  |  |
| Yes | 303 | 804 | 75 |  |  |  |
|  |  |  |  |  |  |  |
| **Health-related factors including herd performance indicators^7^:** | | | | | |  |
| Presence of digital dermatitis in the herd according to farm owner/staff^5^ | | | | Q | 0.08 | 0.174 |
| No | 244 | 928 | 82 |  |  |  |
| Yes | 226 | 593 | 44 |  |  |  |
| Presence of ectoparasites in cows during the last year  according to farm owner/staff | | | | Q | 0.24 | 0.01 |
| No | 473 | 1,171 | 90 |  |  |  |
| Yes | 356 | 1,011 | 116 |  |  |  |
| Group treatment of cows against ectoparasites during the last year  according to farm owner/staff | | | | Q | 0.41 | 0.40 |
| No | 659 | 1,663 | 155 |  |  |  |
| Yes | 170 | 519 | 51 |  |  |  |
| Calf mortality 0 - 24h (%)^7^ | | | | C | 0.78 | 0.99 |
| 0 - 5.9 | 436 | 1,126 | 104 |  |  |  |
| ≥ 6 | 357 | 994 | 99 |  |  |  |
| Calf mortality 1 - 60 days (cases/100 animals at risk)^7^ | | | | C | 0.148 | 0.83 |
| 0 - 2.1 | 387 | 1,127 | 100 |  |  |  |
| ≥ 2.2 | 406 | 993 | 103 |  |  |  |
| Calf mortality 2-6 months (cases/100 animals at risk)^7^ | | | | C | 0.065 | 0.88 |
| 0 - 1.1 | 350 | 1,057 | 86 |  |  |  |
| ≥ 1.2 | 443 | 1,063 | 117 |  |  |  |
| Young stock (6 - 15 months) mortality (cases/100 animals at risk)^7^ | | | | C | 0.56 | 0.80 |
| 0 - 0.09 | 468 | 1,273 | 109 |  |  |  |
| ≥ 0.1 | 325 | 847 | 94 |  |  |  |
| Cow mortality (cases/100 cows at risk)^7^ |  |  |  | C | 0.75 | 0.40 |
| 0 - 4.9 | 420 | 1,119 | 94 |  |  |  |
| ≥ 5 | 373 | 1,001 | 109 |  |  |  |
| Culling of first parity cows in early (0 - 90 days) lactation  (cases/100 cows at risk)^7^ | | | | C | 0.72 | 0.48 |
| 0 - 2.4 | 410 | 1,077 | 94 |  |  |  |
| ≥ 2.5 | 383 | 1,043 | 109 |  |  |  |
| Culling due to udder diseases (cases/100 cows at risk)^7^ | | | | C | 0.31 | 0.97 |
| 0 - 8.9 | 413 | 1,052 | 104 |  |  |  |
| ≥ 9 | 380 | 1,068 | 99 |  |  |  |
| Culling due to hoof- and leg diseases (cases/100 cows at risk)^7^ | | | | C | 0.027 | 0.006 |
| 0 - 2.6 | 414 | 970 | 77 |  |  |  |
| ≥ 2.7 | 379 | 1,150 | 126 |  |  |  |
| Culling due to reproduction diseases (cases/100 cows at risk)^7^ | | | | C | 0.186 | 0.50 |
| 0 - 7.4 | 450 | 1,110 | 111 |  |  |  |
| ≥ 7.5 | 343 | 1,010 | 92 |  |  |  |
| Culling due to any reason (cases/100 cows at risk)^7^ | | | | C | 0.35 | 0.41 |
| 0 - 33 | 412 | 1,028 | 92 |  |  |  |
| ≥ 34 | 381 | 1,092 | 111 |  |  |  |
| Cows with abnormally low (≤ 2 mM/l) milk urea concentration (%)^7^ | | | | C | 0.035 | 0.005 |
| 0 - 6.4 | 368 | 1,171 | 130 |  |  |  |
| ≥ 6.5 | 390 | 871 | 70 |  |  |  |
| Cows with abnormally high (≥ 6 mM/l) milk urea concentration (%)^7^ | | | | C | 0.96 | 0.30 |
| 0 - 6.1 | 390 | 1,024 | 90 |  |  |  |
| ≥ 6.2 | 368 | 1,018 | 110 |  |  |  |
| Cows with abnormal (≤ 2 or ≥ 6 mM/l) milk urea concentration (%)^7^ | | | | C | 0.021 | 0.30 |
| 0 - 15.9 | 367 | 1,154 | 110 |  |  |  |
| ≥ 16 | 391 | 888 | 90 |  |  |  |
| Bulk tank milk SCC (cells/ml)^7^ | | | | C | 0.26 | 0.28 |
| < 242,000 | 385 | 1,106 | 114 |  |  |  |
| ≥ 242,000 | 373 | 936 | 86 |  |  |  |
| Veterinary-treated clinical mastitis (cases/100 cows at risk)^7^ | | | | C | 0.52 | 0.44 |
| 0 - 9.9 | 408 | 1,000 | 95 |  |  |  |
| ≥ 10 | 385 | 1,120 | 108 |  |  |  |
| Veterinary-treated feeding-related diseases (cases/100 cows at risk)^7^ | | | | C | 0.98 | 0.94 |
| 0 - 0.9 | 409 | 1,089 | 103 |  |  |  |
| ≥ 1.0 | 384 | 1,031 | 100 |  |  |  |
| Veterinary-treated hoof- and leg diseases (cases/100 cows at risk)^7^ | | | | C | 0.72 | 0.58 |
| 0 - 0.7 | 398 | 1,046 | 97 |  |  |  |
| ≥ 0.8 | 395 | 1,074 | 106 |  |  |  |
| Veterinary-treated parturient paresis, other types of paresis and hypomagnesemia (cases/100 cows at risk)^7^ | | | | C | 0.57 | 0.83 |
| 0 - 2.1 | 423 | 1,045 | 104 |  |  |  |
| ≥ 2.2 | 370 | 1,075 | 99 |  |  |  |
| All veterinary-treated diseases, including the above  (cases/100 cows at risk)^7^ | | | | C | 0.62 | 0.87 |
| 0 - 21 | 410 | 1,020 | 120 |  |  |  |
| ≥ 22 | 383 | 1,100 | 101 |  |  |  |
| Average age at first calving (months)^7^ | | | | C | 0.77 | 0.56 |
| < 27 | 416 | 1,135 | 100 |  |  |  |
| ≥ 27 | 377 | 985 | 103 |  |  |  |
| Frequency of assisted calvings (%)^7^ | | | | C | 0.047 | 0.034 |
| 0 - 2.6 | 344 | 1,076 | 108 |  |  |  |
| ≥ 2.7 | 449 | 1,044 | 95 |  |  |  |
| Cows with > 70 days between calving and first insemination (%)^7^ | | | | C | 0.97 | 0.67 |
| 0 - 19 | 381 | 1,041 | 109 |  |  |  |
| ≥ 20 | 412 | 1,079 | 94 |  |  |  |
| Cows with > 120 days between calving and final insemination (%)^7^ | | | | C | 0.40 | 0.33 |
| 0 - 6.4 | 398 | 992 | 89 |  |  |  |
| ≥ 6.5 | 395 | 1,128 | 114 |  |  |  |
| Heifers > 17 months not inseminated (%)^7^ | | | | C | 0.005 | 0.031 |
| 0 - 16 | 351 | 1,159 | 116 |  |  |  |
| ≥ 17 | 442 | 961 | 87 |  |  |  |

^1^ Continuous variables were assessed if they were linearly related to the outcome, and if not, they were categorized using percentiles as cut-offs

^2^ The total number of cows for each variable differs due to missing data or if variable categories don’t apply to all herds (e.g. lower front rail absent in many herds)

^3^ I = Information obtained via: O = observed at herd visit, Q = questionnaire, answers obtained via interviewing farm owner or staff, C = data from the Swedish Official Milk Recording Scheme (SOMRS)

^4^ Data from the SOMRS based on individual herd data from the 12 months preceding the visit

^5^ Variable excluded from multivariable analyses due to missing data

^6^ Cows within this category excluded from the multivariable analyses due to few cows/herds within the category

^7^ Herd performance indicators from the SOMRS, based on individual herd data from the 12 months preceding the visit
